# Alkaline Water Mitigates Bone Loss in Streptozotocin-Induced Type II Diabetic Rats

**DOI:** 10.7759/cureus.59833

**Published:** 2024-05-07

**Authors:** Bandar A Alghamdi

**Affiliations:** 1 Department of Surgery, College of Medicine, Umm Al-Qura University, Al-Qunfudhah, SAU

**Keywords:** osteoclast, osteoporosis, osteoblast, zamzam water, diabetes

## Abstract

Background

A decline in bone mineral density is a defining feature of osteoporosis, which is a prevalent bone complication associated with diabetes. This study aims to shed light on the protective effect of Zamzam water (ZW), a famous alkaline water, on diabetes-induced osteoporosis.

Methodology

Of a total of 40 male rats, 10 male rats each were divided into the following four groups: group I (normal control rats), group II (ZW group), group III (diabetic rats), and group IV (DM + ZW). Anteroposterior and lateral X-rays were taken of the rats in each group before the end of the experiment. The study assessed serum levels of inflammatory markers including interleukin 6, interleukin-1 beta, and tumor necrosis factor-alpha; bone formation markers including osteocalcin (OC); alkaline phosphatase (ALP); and bone resorption markers including the N-terminal telopeptide of collagen type I (NTX-1), bone deoxypyridinoline (DPD), and tartrate-resistant acid phosphatase 5b (TRAP-5b).

Results

Rats with diabetes who consumed ZW exhibited a significant (p < 0.001) increase in OC and ALP bone formation markers and a decrease in NTX-1, DPD, and TRAP-5b bone resorption markers, with improvements in the X-ray image of the vertebral column at the L6 vertebra level.

Conclusions

ZW improved diabetes-induced osteoporosis in rats by enhancing osteoblastic activity and downregulating osteoclastic activity.

## Introduction

According to the International Diabetes Federation, 451 million adults between the ages of 18 and 99 were diagnosed with diabetes in 2017, and, by 2045, this figure is predicted to increase to nearly 693 million [1]. Globally, approximately 8-9 million fractures occur annually due to osteoporosis, with one instance occurring every three seconds [2]. Osteoporosis and diabetes both pose major risks to a person’s health and quality of life, particularly for the elderly. This has led to aging societies facing significant public health issues. In 1948, Albright et al. discovered a connection between osteoporosis and diabetes and proposed the theory of diabetic osteoporosis [3]. One of the most serious effects of diabetes mellitus (DM) is diabetic osteoporosis, a systemic endocrine metabolic osteopathy that raises the risk of fractures linked to bone fragility. In addition, various studies have shown that people with type 2 diabetes are significantly more likely to suffer from osteoporosis and fractures than people without the disease [4-6].

Previous studies have demonstrated the potential significance of several factors in the pathophysiological process of diabetic osteoporosis, including insulin resistance, oxidative stress, chronic hyperglycemia, insulin-like growth factor 1, and the use of hypoglycemic medications [7-9].

In addition to the oxidative stress caused by diabetes, another mechanism plays a role in bone loss by increasing osteoclast activity and subsequently leading to more bone resorption than bone formation, such as gouty arthritis, inflammation [10], and vitamin D deficiency [11].

Because of osteoporosis, bones become so fragile and feeble that they easily break with little energy. Typically, the wrist, hip, or vertebrae are affected by these osteoporosis-related fractures [12]. A few recent studies have shown that various animal models, including rat, pig, dog, and sheep models, can be used to model vertebral fractures and their associated characteristics [13].

Many bone resorbing markers, including tartrate-resistant acid phosphatase 5b (TRAP-5b), deoxypyridinoline (DPD), and N-terminal telopeptide of collagen type I (NTX-1), as well as bone-forming cytokines, such as procollagen type 1 N-terminal propeptide and alkaline phosphatase (ALP), bone-specific ALP or osteocalcin (OC), are released during the process of bone remodeling [14].

Zamzam water (ZW) is naturally endowed with properties that render it alkaline, characterized by a significant content of vital mineral salts, thereby providing a substantial energy boost to the body. Additionally, it acts as a neutralizing agent for pH, confers protection against diseases, and exerts potent antioxidative effects [15]. It has been found that ZW possesses several essential minerals that are necessary for maintaining optimal bone health [16]. This study aims to clarify the role that the well-known alkaline water ZW plays in preventing osteoporosis caused by diabetes.

## Materials and methods

Animals

A total of 40 Sprague-Dawley male rats weighing between 200 and 240 g were housed in an experimental laboratory with a 12-hour day-night cycle. The rats were kept in an experimental environment with 60-80% humidity and 22-26°C temperature. All experimental protocols were conducted under the supervision of the Biomedical Research Ethics Committee, Umm Al-Qura University, Makkah, Saudi Arabia (approval number: HAPO-02-K-012-2023-06-1663).

Experimental animals and treatment

Before the first week of the experiment, the rats were housed to accommodate the experimental environment. The 40 rats were divided into the following four different experimental groups: group 1 (control group; n = 10) rats were fed a normal diet, drank 0.9 mL of normal saline, and were injected with citrate buffer as a solvent; group II (ZW group; n = 10) rats received a normal diet and ZW 100 mL/cage; group III (diabetic group; n = 10) rats were fed a high-fat diet consisting of 37 kcal% fat, 46 kcal% carbohydrates, and 17 kcal% protein (4.40 kcal/g of food) for four weeks; and group IV (diabetic + ZW; n = 10) diabetic rats received ZW at a dose of 100 mL/cage. Subsequently, an intraperitoneal injection of streptozotocin (Sigma, USA) at a dose of 35 mg/kg body weight was administered to rats. After 72 hours, the rats were kept fasting for eight hours. After that, the rat’s tail vein samples were obtained, and fasting blood sugar was measured. Rats that tested higher than 11 mmol/L for fasting blood glucose were classified as diabetic and included in the study [17]. After two months, the rats were harvested, and blood samples were stored at -80°C for biochemical and enzyme-linked immunosorbent assay (ELISA) examinations.

Assessment of vertebral column osteoporotic changes by X-ray

The rats in the various study groups were anesthetized before the end of the experiment and their vertebral column was photographed by an X-ray scanner (AXIOM Aristos AX/TX/VX; Siemens, Germany) in anteroposterior and lateral views. The rats were placed in a prone position on the X-ray machine platform, with L6 serving as the center of the image. L6 can be easily defined as the site of the intersection of the high point of the iliac crest with the spine. Sagittal radiograph images were acquired at 50 kVp, 6.2 mAs, and 50 ms.

Biochemical parameters

ELISA kits were utilized to measure the serum levels of NTXI (MBS2700254), TRAP-5b (MBS2702692), DPD (MBS2506789), OC (MBS2701838), ALP (MBS312841), interleukin 6 (IL-6) (MBS2021530), interleukin-1 beta (IL-1β) (MBS650942), and tumor necrosis factor (TNF-α) (MBS175820) according to the manufacturing rules.

Statistical analysis

The study data were analyzed using GraphPad Prism version 8.0 (San Diego, CA, USA). All data were expressed as mean ± SD. A one-way analysis of variance was used to compare the outcomes of the various groups, and a post-hoc Tukey honestly significant difference test was used to determine significance if the p-value was less than 0.05.

## Results

Ameliorative effect of ZW on vertebral column osteoporotic and diabetic-induced changes

**Figure 1:**
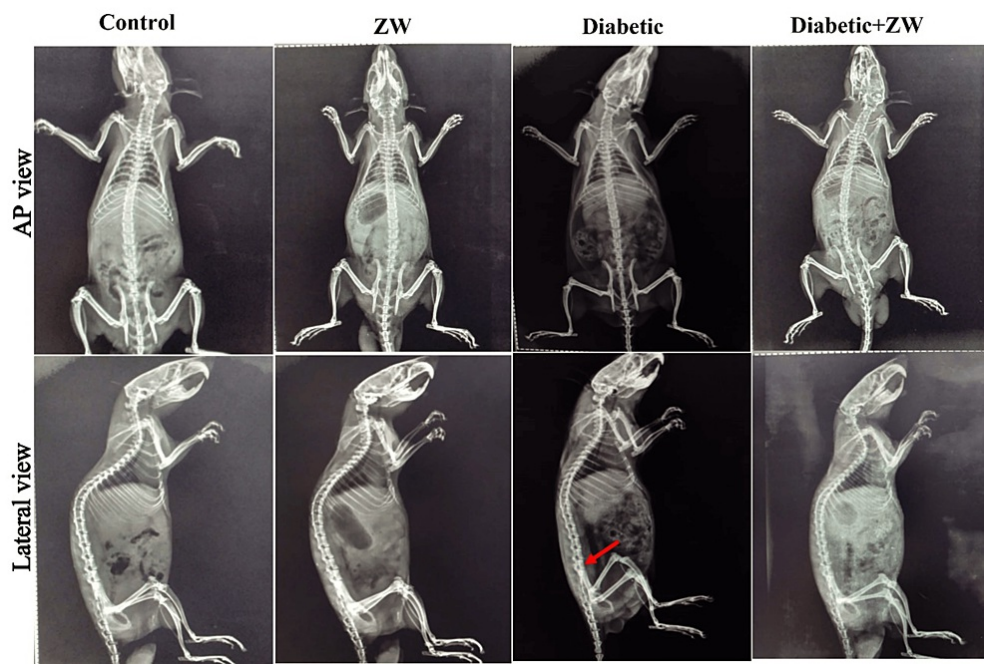
Anteroposterior and lateral view of the rat’s vertebral column from an X-ray taken of each group. The diabetic group’s lateral view shows bone defects at the L6 level, as indicated by the red arrow.

As shown in Figure 1, the diabetic rats group showed vertebral column bone defects at the L6 vertebra level, as shown either in sagittal plain or lateral X-ray, compared to the control and negative control groups. In contrast, drinking ZW at a dose of 100 mL/cage showed improvement in plain and lateral X-rays at the L6 level in comparison to the diabetic group. From this result, we can conclude that ZW offers a protective effect against diabetes-induced osteoporotic changes.

Ameliorative effect of ZW on serum inflammatory markers IL-6, IL-1β, and TNF-α in streptozotocin-induced diabetic rats

**Figure 2:**
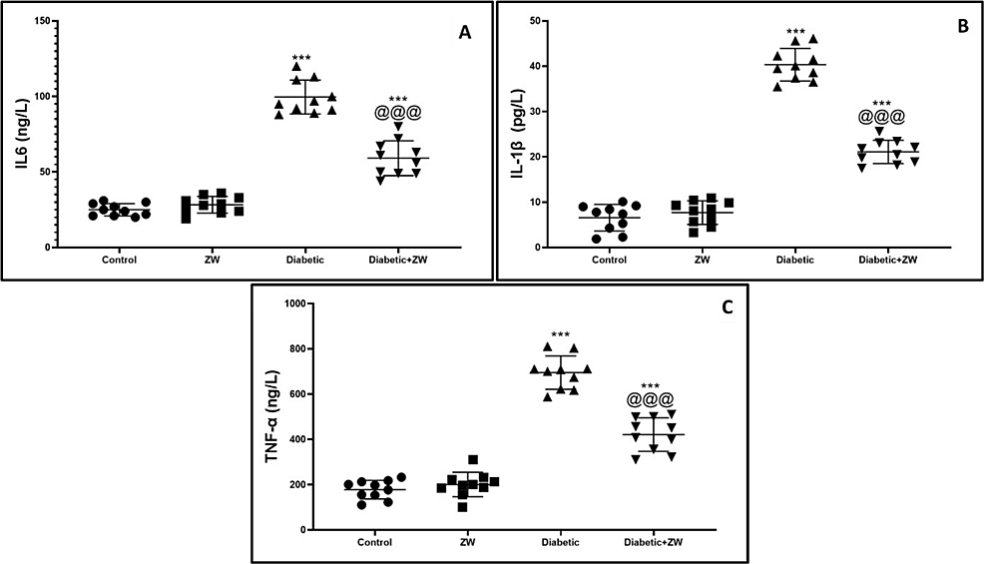
Impact of ZW on the levels of inflammatory markers in serum. (A) IL-6, (B) IL-1β, and (C) TNF-α. ***: p < 0.001 versus control; @@@: p < 0.001 versus diabetic group. IL-6 = interleukin 6; IL-1β = interleukin-1 beta; TNF-α = tumor necrosis factor-alpha; ZW = Zamzam water

ELISA examination of serum levels of inflammatory markers in diabetic rats discovered a significant (p < 0.001) elevation in IL6, IL-β, and TNF-α by 99.6 ± 11.22, 40.31 ± 3.60, and 695.2 ± 73.84, respectively, in comparison to control rats. On the contrary, the diabetes + ZW group showed a marked decrease in the level of inflammatory mediators by 59.10 ± 11.59, 21.9 ± 2.56, and 421.5 ± 74.21, respectively, in comparison to the diabetic group (Figures 2A-2C). From the results, we can explore the anti-inflammatory effect of ZW in diabetic rats.

Protective effect of ZW on bone resorption markers NTXI, DPD, and TRAP-5b and bone formation markers OC and ALP in streptozotocin-induced diabetic rats

**Figure 3:**
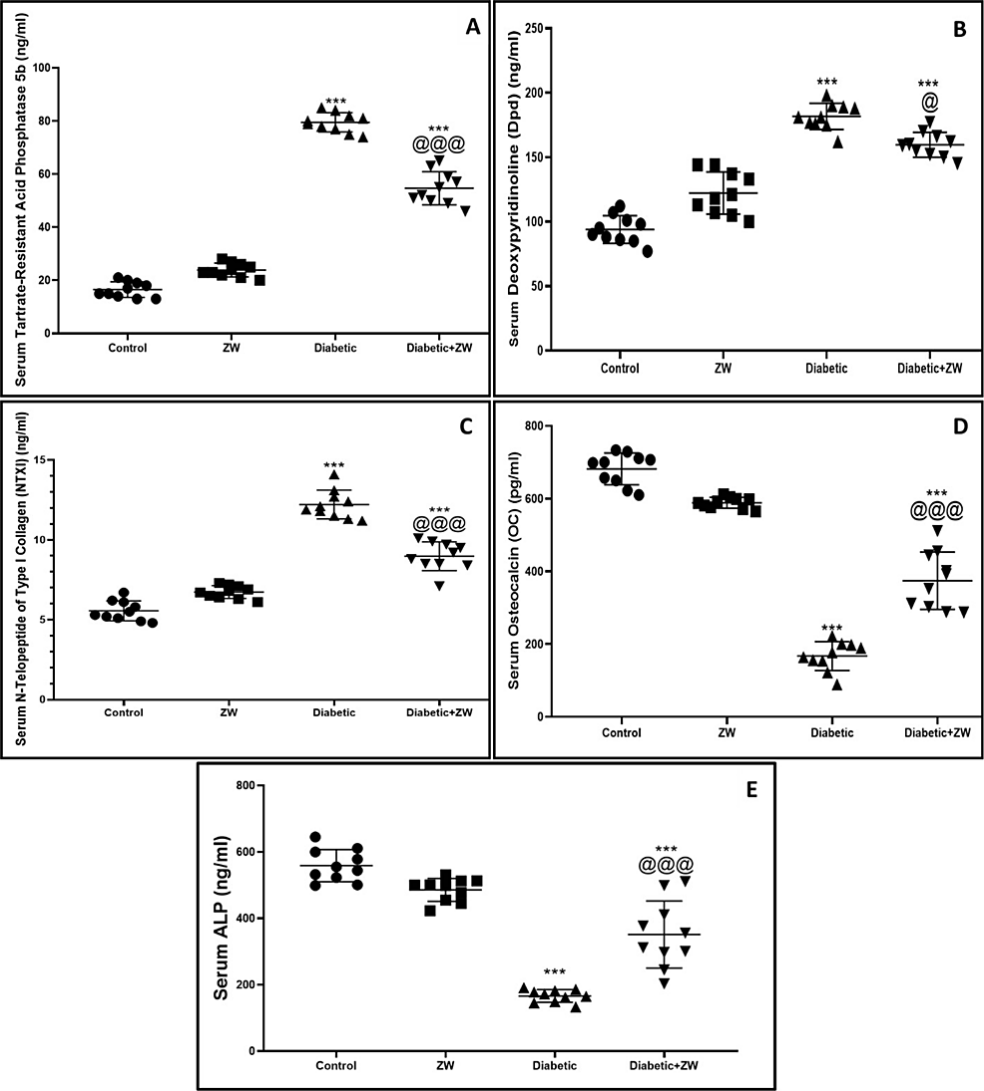
Effect of ZW on the levels of bone resorption markers. (A) DPD, (B) TRAP-5b, (C) NTXI, (D) OC, and (E) ALP. ***: p < 0.001 versus control; @@@: p < 0.001 versus diabetic group. DPD = deoxypyridinoline; TRAP-5b = tartrate-resistant acid phosphatase 5b; NTXI = N-terminal telopeptide of collagen type I; OC = osteocalcin; ALP = alkaline phosphatase; ZW = Zamzam water

To determine how diabetes affects bone loss, we evaluated the serum levels of NTXI, DPD, and TRAP-5b. Furthermore, for bone formation, we measured OC and ALP bone formation markers using an ELISA assay. We found that diabetes significantly (p < 0.001) increased the serum levels of NTXI, DPD, and TRAP-5b bone resorption markers by 12.21 ± 0.89, 181.7 ± 10.09, and 79.50 ± 2.60, respectively, and at the same time decreased bone formation markers OC and ALP by 167.2 ± 39.63, 166.3 ± 19.14, respectively, in comparison to the control group. Interestingly, drinking ZW in the diabetes + ZW group significantly (p < 0.001) decreased the serum levels of NTXI, DPD, and TRAP-5b bone resorption markers by 8.97 ± 0.90, 159.6 ± 9.67, and 54.70 ± 6.23, respectively, and increased the serum levels of OC and ALP bone formation marker by 374.3 ± 78.80, 351.3 ± 101, respectively, in comparison to the diabetic group (Figures 3A-3D). 

## Discussion

This study was conducted to demonstrate the benefits of using ZW on bones, particularly in diabetic osteoporosis. One of the most common complications of diabetes is a reduction in bone mineral density, which results in bone fractures [18]. Several studies have discussed the fragility of bones and their liability to fracture due to an elevation in blood sugar [19-21]. Diabetic patients mostly suffer from bone loss with an increase in bone turnover [22]. Bone is characterized by its dynamic structure. It is in continuous formation and breakdown, which is known as bone remodeling. This process is regulated by the balance between osteoblast and osteoclast cells. Unfortunately, diabetes impairs osteoblast differentiation, which negatively affects bone formation and mineralization [23]. It also induces apoptosis of osteoblastic cells, which explains diabetic-induced neutropenia [24,25].

The following results were noted in this study: (a) elevated blood sugar in rats resulted in a significant elevation in inflammatory cytokines (IL6, IL-1β, and TNF-α) and bone resorption markers (TRACP-5b, DPD, and NTXI) with significant depression in bone formation markers (OC and ALP); (b) defect in the L6 vertebra on anteroposterior and lateral X-rays in diabetic rats; and (c) diabetic rats drinking ZW for eight weeks had significantly decreased inflammatory and bone resorption markers as well as increased bone formation markers.

Elevation of proinflammatory cytokines in bone tissue increased the number of osteoclast cells [26] due to a decrease in osteoclast apoptosis and upregulation of osteoclast precursor cells with their subsequent differentiation into mature osteoclast [27]. In this study, ELISA examination of diabetic rats’ serum revealed a significant elevation of proinflammatory markers, consistent with a prior study by Lu et al. [17]. When ZW was consumed by diabetic rats, the levels of inflammatory cytokines significantly decreased. The results of this study are consistent with those of Taha et al. [28], who demonstrated the anti-inflammatory properties of ZW in reducing testicular inflammation caused by gentamicin.

The microarchitecture of bone and bone mineral density were used as indicators to assess bone quality [29,30]. Osteoporosis of the vertebral column is one of the most dangerous complications of osteoporosis, increasing the risk of disability [31]. Bone turnover biomarkers include bone formation and resorption markers [32]. Osteogenic markers OC and ALP are synthesized by osteoblasts, which reflect bone formation [33].

TRAP-5a is one of the bone-resorbing markers secreted by activated osteoclasts [34]. Furthermore, NTXI is an indicator of bone resorption [35]. Moreover, DPD, one of the bone resorption markers, results from collagen decomposition that occurs during bone turnover [36].

Our study revealed that diabetic rats showed a significant rise in markers of bone resorption (TRACP-5b, DPD, and NTXI) and a decrease in bone formation markers (OC and ALP), which manifested in the form of L6 vertebra bone defects. This finding is parallel to the study by Lu et al. [17], which documented a decrease in OC and ALP bone formation markers. Another study done by Alrowaili et al. [37] demonstrated elevations in TRAP-5b, NTX-1, and DPD bone resorption markers in glucocorticoid-induced osteoporosis. On the contrary, diabetic rats receiving ZW significantly increased osteoblastic markers (OC and ALP), while at the same time decreasing bone resorption markers (TRAP-5b, NTX-1, and DPD), with clear improvement in the X-ray image of the L6 lumbar vertebra level. This can be regarded as the physical and chemical characteristics of ZW due to its alkaline composition and its high concentration of trace elements, such as magnesium (520 mg/L) and calcium (189 mg/L), in comparison to tap water, which contributes to bone density, as well as its powerful antioxidant character by elevating antioxidant enzymes [38]. Oxidative stress markedly inhibits osteoblast differentiation and activation [39]. Also, the anti-inflammatory effect of ZW inhibits osteoblastic differentiation and bone resorption, with a decrease in bone resorption markers.

The strength of the study is that it is the first to discuss the effect of alkaline water on diabetic osteoporosis and uses ZW as a natural therapy without chemical compounds, while the limitation is the small sample size, as rats can easily die due to diabetes. This study’s limitations can be summed up as follows: the small sample size, radiographs were not obtained before the start of the experiments, and the study was based on an animal model rather than humans; however, rats and humans are similar histologically. In addition, we did not measure bone density using the dual X-ray absorptiometry bone scan because it was not available. Moreover, some antibodies, such as the RANKL index of osteoclast osteoclastogenesis, were not available. These points will be considered in future work.

## Conclusions

We can conclude that diabetes raises the serum levels of the bone resorption markers TRAP-5b, NTX-1, and DPD while dramatically lowering those of the bone formation markers OC and ALP, along with a vertebral defect at the level of the sixth lumbar vertebra of a rat. On the other hand, eight weeks of ZW intake by diabetic rats improved the osteoporotic X-ray appearance of the spine, probably through the activation of osteoblasts and inhibition of osteoclast cells. The preclinical findings of this study can guide future clinical research on the effectiveness of ZW, a safe natural alkaline water, in treating diabetic osteoporosis.
